# Meeting report: mobile genetic elements and genome plasticity 2018

**DOI:** 10.1186/s13100-018-0126-3

**Published:** 2018-06-22

**Authors:** John M. Abrams, Irina R. Arkhipova, Marlene Belfort, Jef D. Boeke, M. Joan Curcio, Geoffrey J. Faulkner, John L. Goodier, Ruth Lehmann, Henry L. Levin

**Affiliations:** 1 University of Texas Southwestern Medical Center, Dallas, TX 75390 USA; 2 Josephine Bay Paul Center for Comparative Molecular Biology and Evolution, Marine Biological Laboratory, 7 MBL St, Woods Hole, MA 02543 USA; 3 Department of Biology, University at Albany, 1400 Washington Avenue, Albany, NY 12222 USA; 4 Institute for Systems Genetics, NYU Langone Health, New York City, NY 10016 USA; 5 Wadsworth Center, New York State Department of Health, Albany, NY 12201 USA; 6 Mater Research Institute – University of Queensland, TRI Building, Woolloongabba, QLD 4102 Australia; 7 McKusick-Nathans Institute for Genetic Medicine, Johns Hopkins University School of Medicine, Baltimore, MD 212051 USA; 8 Howard Hughes Medical Institute and Kimmel Center for Biology and Medicine of the Skirball Institute, Department of Cell Biology, New York University School of Medicine, New York, NY 10016 USA; 9 Eunice Kennedy Shriver National Institute of Child Health and Human Development, National Institutes of Health, Bethesda, MD 20892 USA

## Abstract

The Mobile Genetic Elements and Genome Plasticity conference was hosted by Keystone Symposia in Santa Fe, NM USA, February 11–15, 2018. The organizers were Marlene Belfort, Evan Eichler, Henry Levin and Lynn Maquat. The goal of this conference was to bring together scientists from around the world to discuss the function of transposable elements and their impact on host species. Central themes of the meeting included recent innovations in genome analysis and the role of mobile DNA in disease and evolution. The conference included 200 scientists who participated in poster presentations, short talks selected from abstracts, and invited talks. A total of 58 talks were organized into eight sessions and two workshops. The topics varied from mechanisms of mobilization, to the structure of genomes and their defense strategies to protect against transposable elements.

## Introduction

Transposable elements (TEs) constitute a major portion of genomes, particularly in eukaryotes, and by causing mutations, rearrangements, and duplications, they have a dramatic impact on genome content. In addition to the importance of TE-mediated mutations that result in disease, there is increasing significance in the role TEs play in shaping expression of regulatory networks. Recent discoveries regarding the function of TEs motivated Keystone Symposia to host the conference on Mobile Genetic Elements and Genome Plasticity in Santa Fe, NM, USA, February 11 through February 15th. Topics discussed at the conference often relied on advances in DNA sequencing and in the analysis of highly repetitive genomes. The presentations described the potent impact of TEs on genetic variation and introduced mechanisms responsible for structural variation in the evolution of primate genomes. Other talks described the discovery of cellular systems that inhibit TE activity, adding new insight to the evolutionary arms race between mobile DNA elements and their hosts. Also included was new evidence of TE activity in neurons and cancer cells. The conference established relationships between scientists working on TE biology, genome evolution, and structural variation. The TEs and hosts discussed at the meeting included a range of systems such as eubacteria, protists, plants, fungi, and animals. The keynote address was given by one of the leaders in developing groundbreaking applications of clustered regularly interspaced short palindromic repeat (CRISPR).

## Keynote address

Keynote Speaker, Feng Zhang (MIT, USA), launched the meeting with a bang. Zhang is well known for developing powerful molecular technologies. He spoke about mining microbial diversity of CRISPR-Cas systems. After using zinc finger and TALE endonucleases to perform genome editing, his lab turned to CRISPR because of its versatility, efficiency and specificity. First, they made a Cas9 enzyme available from *Staphylococcus aureus* (SaCas9), whose gene is more than 1 kb shorter than the original *S. pyogenes Cas9* homolog. The shorter SaCas9 is better accommodated by adeno-associated virus (AAV) vectors. They packaged SaCas9 and guide RNA into AAV to target the DNA of *Pask9*, a cholesterol regulatory gene in mammalian cells, to yield significant decreases in cholesterol levels.

In collaboration with the Koonin lab, Zhang explored CRISPR diversity in different bacterial systems, leading to the discovery of the Cas13 RNA targeting system. Cas13 has a curious collateral activity that degrades RNA non-specifically once the CRISPR Cas13 system has recognized the RNA target. In a clever design, this collateral effect of Cas13 was harnessed to cleave reporter RNA and release signal (e.g. fluorescence), to provide rapid DNA or RNA detection with high sensitivity and specificity. This specific high-sensitivity enzymatic reporter unlocking (SHERLOCK) system has applications in pathogen detection, both viral (Zika and Dengue) and bacterial, in DNA genotyping and in mutation detection in tumors, and has been adapted for use in the field. The method can be multiplexed for the simultaneous detection of different nucleic acids. In a second application, catalytically inactive Cas13 was used to direct adenosine-to-inosine deamination by ADAR2, to edit transcripts in mammalian cells with damaging mutations. The sophisticated combination of technologies made the talk a tour de force!

## Mechanisms and results of genome editing (Laura Landweber, session chair)

Mitchell O’Connell (University of Rochester, USA) described his work understanding the molecular mechanisms of Cas13 that protects a range of bacterial species from TEs. Specifically, he described biochemical and structural data that showed Cas13 contains two distinct RNA-nuclease modalities: one nuclease activity required to cleave foreign phage mRNA and the other activity required to generate mature guide-RNAs from long precursors. As described by Zhang, O’Connell also showed that these RNA activities can be utilized to create a sensitive RNA detection assay to measure RNA abundances within complex samples. O’Connell finished by describing unpublished observations regarding the specificity by which Cas13 binds and cleaves its RNA targets.

John Schiel (Horizon Discovery, USA) discussed precise genome repair with the CRISPR-Cas9 system using the homology-directed repair (HDR) pathway. The efficiency of gene knock-in with the HDR pathway is much lower when compared to gene knockout with the NHEJ pathway. Schiel compared different formats of repair templates, such as ssDNA and plasmid, to evaluate the ability of each repair template to knock-in EGFP at the same position in the gene target, using the same synthetic guide RNA. Design and experimental conditions of each template were evaluated to provide optimal recommendations for template and homology arm length.

Zoltan Ivics (Paul Ehrlich Institute, Germany) provided an update on the use of the TE Sleeping Beauty as a vector for gene therapy. There are several clinical trials already running and additional clinical applications are approaching to treat diseases of the eye and the hematopoietic system that rely on TE-mediated gene delivery. One fundamental issue with gene therapies is potential side effects elicited by vector integration. Ivics reported on a comparative insertion site profiling of the TEs Sleeping Beauty and piggyBac as well as gammaretroviral and lentiviral vectors. Unlike the other genetic elements in this study, Sleeping Beauty appears to lack a preference for gene sequences, suggesting a potential for enhanced safety in a clinical context.

Josh Dubnau (Stony Brook University, USA) described TDP-43, an RNA and DNA binding protein that forms cytoplasmic inclusions in the vast majority of both familial and sporadic cases of amyotrophic lateral sclerosis (ALS). Using a Drosophila TDP-43 over-expression model, Dubnau has recapitulated characteristics of TDP-43-linked disease including protein aggregation and locomotor impairment. Such expression results in loss of siRNA silencing and de-repression of both LINE and long terminat repeat (LTR) families of retrotransposable elements (RTEs). The RTE activity contributes to the degenerative phenotypes in these flies. Dubnau established that TDP-43 toxicity in glial cells involves a fundamental non-cell autonomous mechanism leading to programmed cell death of both glial cells and adjacent neurons. Similar to the human endogenous retrovirus K (HERV-K), which is expressed in the cortex of some ALS subjects, the gypsy RTE retains a functional *env* gene, which provides the potential for non-cell autonomous transfer to adjacent neurons. In co-culture experiments, Dubnau found that gypsy elements are indeed able to transfer reporter expression. Dubnau’s findings raise the possibility that HERV-mediated movement between cells could contribute to the focal onset and spread of neurodegenerative disorders within the nervous system.

Laura Landweber (Columbia University, USA), introduced the ciliate *Oxytricha trifallax,* which undergoes massive DNA rearrangements that produce a highly fragmented but functional somatic macronucleus from a complex germline micronucleus. Landweber discussed how this process eliminates nearly all noncoding DNA, including transposons, and rearranges over 225,000 short DNA segments to produce a second genome containing thousands of gene-sized “nanochromosomes.” She also showed data indicating that noncoding RNAs regulate the process of genome rearrangement, including millions of 27 nt piRNAs that mark and protect the retained DNA segments. She finished by describing how maternally-inherited, long, non-coding (lnc) RNAs also serve as templates for genome remodeling and RNA-guided DNA repair.

## Workshop 1: Evolutionary mechanisms of transposition (Irina Arkhipova, session chair)

Hyo Won Ahn (Garfinkel lab, University of Georgia, Athens, USA) studied the copy number control (CNC) of the Ty1 LTR retrotransposon in yeast. A group of genes required for ribosome function was found to modulate the ratio of the self-encoded restriction factor p22 (a truncated Gag) to its target, the capsid protein Gag. Importantly, Ty1 virus-like particle assembly and function was inhibited in cells lacking the ribosome biogenesis factor Loc1, revealing an unexpected relationship between ribosome biogenesis and Ty1 CNC.

Agnes Michel (Kornmann lab, ETH Zurich, Switzerland) introduced SAturated Transposon Analysis in Yeast (SATAY), a TE-based method that functionally explores the *Saccharomyces cerevisiae* genome. Akin to the Transposon Mutagenesis followed by Deep Sequencing (Tn-seq) approach in bacteria, it is based on the generation of a dense library of TE insertion mutants (here, TE is the Ac/Ds transposon from maize). Insertion sites are identified in bulk by deep sequencing and mapped onto the genome. Because TE insertions in essential genes kill the host, such genes or even their sub-regions appear as TE-free. Repeating the procedure in different conditions (e.g. drug treatment or different genetic background) uncovers sets of genes important for growth in a condition-specific manner.

Irina Arkhipova (Marine Biological Laboratory, Woods Hole, USA) presented evidence that a horizontally transferred bacterial methyltransferase, which in prokaryotes typically encodes a component of the restriction-modification system, was recruited to deposit non-canonical epigenetic marks into genomic DNA of bdelloid rotifers, known for their ability to acquire foreign genes. Many TEs and other repeats display an elevated density of epigenetic marks, and the recombinant enzyme was shown to exhibit methyltransferase activity, possibly providing an additional layer of TE suppression to a multi-layered genome defense system in these tiny freshwater invertebrates.

Shunhua Han (Bergman lab, University of Georgia, Athens, USA) investigated the process of TE proliferation in Drosophila S2 tissue culture cells by sequencing the genomes of 25 S2 sub-lines and reconstructing the evolutionary history of TE insertion in multiple S2 cell lineages. He showed that TE insertion in S2 cells is an ongoing process that is driven by a limited number of TE families and may substantially affect gene structures. Thus, the S2 cell lines used by different laboratories may be expected to have different genome organization and/or different functional properties.

Arnab Ghosh (Ray lab, Texas Tech University, USA) characterized miRNA and piRNA pools in the saltwater crocodile, *Crocodylus porosus*. Crocodilians are known to evolve very slowly, with a very low rate of TE accumulation. The miRNAs were primarily associated with type II TEs (DNA TEs), while the piRNAs displayed a characteristic ping-pong signature and were mostly associated with type I retrotransposons (LINEs), followed by DNA TEs. These findings provide the first insights into the evolutionary impact of miRNAs and piRNAs in reptiles and their possible role in regulation of TEs in the crocodilian genome.

Jasmine Baker (Batzer lab, Louisiana State University, USA) identified 46 lineage-specific Alu subfamilies in the squirrel monkey (Saimiri), a New World monkey commonly used in biomedical research. Retrotransposition activity involved subfamilies related to AluS, AluTa10, and AluTa15. Of the 110 Alu insertion polymorphisms, 51 had species-informative allele frequency distributions between *Saimiri sciureus* and *Saimiri boliviensis* groups. Thus, Alu elements are undergoing prolific expansion in Saimiri, with many active subfamilies propagating concurrently.

Sung-Yeon Hwang (Ahn lab, Seoul National University, South Korea) reported that RNaseH2, a DNA-RNA hybrid-specific enzyme, is a binding partner of MOV10, a superfamily I RNA helicase previously shown to suppress LINE-1 retrotransposition. Interplay between RNaseH2 and MOV10 is required for inhibition of human LINE-1 mobility, and the loss of MOV10 or RNaseH2 results in accumulation of LINE-1-derived RNA-DNA hybrids, i.e. intermediates of target-primed reverse transcription. Finally, MOV10 and RNaseH2 were shown to control synovial inflammation through suppressing LINE-1 mobility, suggesting a link between L1 restriction and the progression of rheumatoid arthritis, an autoimmune disease.

## Dynamics of plasticity (John Abrams, session chair)

Marlene Belfort (University at Albany, USA) described mobile self-splicing introns and inteins as stress sensors. She discussed how sequence similarity networks are used to understand how these elements are disseminated in nature. Most remarkably, inteins can respond to different stresses and engage in instantaneous splicing only under ideal conditions for protein function. Thus, these self-splicing elements not only contribute to genome plasticity as mobile genetic elements, but also have adapted to become environmentally responsive stress sensors.

Ling-Ling Chen (Shanghai Institutes for Biological Sciences, China) showed that Alu elements facilitate biogenesis of circular RNAs (circRNA) via exon back splicing. Using genome-scale screens, she discussed NF90/NF110 as a key factor important for circRNA biogenesis. She further showed how viral mRNAs in cytoplasm recruit NF90/NF110 from the nucleus, thereby decreasing circRNA production.

Orsolya Barabas (European Molecular Biology Laboratory, Germany) presented work from her group on Tn1549 transposition, which drives the transfer of resistance genes in bacterial populations. Using elegant reconstitution assays, they generated high-resolution structures of transposase complexed with excised circular transposon DNA intermediates, revealing specific steps, DNA distortions, and cleavage mechanisms that enable DNA strand exchange with a minimum of homology.

David Walker (Harshey lab, University of Texas at Austin, USA) used transposable bacteriophage Mu to map genomic organization in *Escherichia coli*. His work revealed a far more dynamic and interactive chromosome than was previously appreciated and established evidence for distal gene clustering.

John Abrams (University of Texas Southwestern Medical Center, USA) described how p53 genes restrict retrotransposon activity throughout the animal kingdom. His group showed that TE eruptions occurring in the p53^−^ germline are incited by meiotic recombination and also detected unrestrained retrotransposons in p53-driven mouse and human cancers. He proposed that ancestral functions of p53 operate to contain retrotransposons, possibly by recruiting repressive epigenetic marks. Since human p53 mutants are disabled for this activity, he raised the possibility that p53 mitigates oncogenic disease in part by restraining transposon mobility.

## Silencing mobile DNA activity: Self and non-self recognition (Brenda Bass, session chair)

R. Keith Slotkin (Ohio State University, USA) presented an update on the “*EpiTEome*” software his lab generated, now published, that enables split-read detection of TE insertion sites from bisulfite-converted DNA methylome data. *EpiTEome* reduces the requirements to perform whole-genome scale TE insertion site detection, as new or existing genome-wide MethylC-seq datasets can be used to detect de novo TE insertion events. Slotkin then presented a case-study of how *EpiTEome* was used in his laboratory to understand dynamic TE activation in plant tissue culture.

Mikiko Siomi (University of Tokyo, Japan) discussed PIWI-interacting RNAs (piRNAs) that bind PIWI proteins to control TEs and maintain germline genome integrity. While Zucchini (Zuc) endonuclease and Nibbler (Nbr) 3′-to-5′ exonuclease play pivotal roles in *Drosophila* piRNA biogenesis, their role in *Bombyx* remains elusive. Siomi showed that loss of Zuc in *Bombyx* caused accumulation of piRNA intermediates. Both 5′ and 3′ ends of piRNA intermediates showed the hallmarks of PIWI-Slicer, yet no phasing pattern was observed in mature piRNAs. Loss of Zuc hardly disturbed the 5′ and 3′ end formation of the intermediates, strongly supporting the notion that the 5′ end of *Bombyx* piRNA is formed by PIWI-Slicer, but independently of Zuc, while the 3′ end is formed by Zuc endonuclease.

Shiv Grewal (NCI, National Institutes of Health, USA) has shown previously that distinct histone methylation patterns organize chromosomes into “open” euchromatin (H3K4me) and “closed” heterochromatin (H3K9me) domains, to modulate use of the genome. He discussed results showing that RNA processing factors and heterochromatin machinery are part of an adaptive cellular mechanism that can reprogram the genome in response to different growth conditions. In particular, work from the Grewal lab suggests that Clr4/Suv39h histone methyltransferase is part of a rheostat-like mechanism, in which transcriptional up-regulation of mRNAs in response to environmental change provides feedback to prevent their uncontrolled expression through heterochromatin assembly. Interestingly, proper iron homeostasis is required, as depletion of iron or down-regulation of iron transporters caused defects in heterochromatin assembly and unrestrained up-regulation of genes. Other results identified connections between RNAi and heterochromatin assembly, which are critical for silencing of repetitive DNA elements including retrotransposons.

Cameron Howard Lee (Wysocka lab, Stanford University, USA) described the use of genome-wide CRISPR/Cas9 screens to identify regulators of L1Hs retrotransposition in human cells. Screening with an L1Hs-G418 reporter in two different cancer cell lines, K562 and HeLa, he identified members of the HUSH complex as the strongest suppressors of retrotransposition. In addition, he also identified many DNA repair factors as being involved in suppressing L1 mobilization, including members of the Fanconi Anemia complex and genes involved in homologous recombination. Surprisingly, knocking out members of the HUSH complex had a large effect on the L1Hs reporter retrotransposition levels but had no effect on a codon-optimized L1Hs reporter, suggesting that the HUSH complex may recognize a particular sequence feature of L1 s. Taken together, Lee’s screens identify many candidate regulators of L1 retrotransposition in human cells and will provide a rich resource for future studies of host regulation of L1 activity.

Todd Macfarlan (NICHD, National Institutes of Health, USA) presented evidence from a ChIP-seq screen of mouse KRAB-ZFPs that showed the majority of mouse KRAB-ZFPs bind to distinct families and sub-families of endogenous retroviruses and LINE1 elements via specific DNA binding motifs. Macfarlan also provided evidence that knocking out KRAB-ZFP gene clusters using a newly developed CRISPR/Cas9 approach in embryonic stem cells leads to predictable activation of ERV expression and nearby genes. He finished with data based on deep sequencing of the genome of mouse tail DNA that the MMETn family of LTR elements increases in copy number in KRAB-ZFP knockout mice.

Brenda L. Bass (University of Utah, USA) explained that adenosine deaminases acting on RNA (ADARs) are a family of enzymes that convert adenosine to inosine in double-stranded RNA (dsRNA). Bass described using ADAR editing sites in high-throughput analyses to create maps of the locations of expressed, long, cellular dsRNA. Not surprisingly, many of these Editing Enriched Regions (EERs) that form long rod-like dsRNA are repetitive elements. In mammals, ADAR editing sites mark dsRNA as self, to distinguish it from viral dsRNA. Consistent with the idea that ADARs mark dsRNA to preclude its entry into the antiviral pathway, Bass finds that *C. elegans* lacking ADARs have siRNAs that map to EERs. EER-associated genes (EAGs) were down-regulated in *adr-1;adr-2* embryos, and this was dependent on associated EERs and the RNAi factor RDE-4. Thus, as in mammals, data from the Bass lab suggests that ADARs mark dsRNA as self in *C. elegans*. At the end of her talk Bass proposed that one role of ADARs is to alleviate silencing of repetitive elements when they inhabit an essential gene.

## The action of mobile DNA in the brain and in Cancer (Geoffrey Faulkner, session chair)

Haig Kazazian (Johns Hopkins University School of Medicine, USA) applied a single-cell targeted L1 sequencing approach to a colorectal tumor, matched metastasis and normal liver. They identified and PCR validated 14 somatic L1 insertions in one patient, including 5 events previously detected by L1 sequencing applied to bulk gDNA extracted from the same tumor. Nearly all of these insertions carried target primed reverse transcription (TPRT) hallmarks and, of those insertions resolved at their 5′ end, about 50% involved an inversion. Interestingly, no PCR validated insertions were found only in normal liver cells. The majority of tumor-specific L1 insertions were found in only one cell each, while the remainder were likely clonal in the tumor. These results highlighted L1-driven heterogeneity during tumor growth and metastasis.

Kathleen Burns (Johns Hopkins University School of Medicine, USA) described L1 activity in pancreatic, ovarian and colorectal tumors. This work identified L1-mediated mutations via targeted sequencing of bulk DNA, and associated L1 retrotransposition with L1 ORF1p and ORF2p expression in patient tissue samples. Immunostains indicated L1 ORF1p expression was increased in malignant tissues as compared to normal, and that this is an early change in cancer precursor lesions. To explore host factors that affect cellular fitness in L1-expressing cells, they conducted genome-wide CRISPR knockout screens and identified novel determinants of cell growth in the presence of L1 expression. These results support a model for L1 involvement in cancer, where L1 may promote the effects of tumor suppressor gene mutations and create specific molecular vulnerabilities in malignant cells.

Patricia Goerner-Potvin (Bourque lab, McGill University, Canada) reported Detecting Repeat Insertions in Long Reads (DRILR), an exciting method able to detect novel TE insertions in PacBio sequencing data. When applied to >30X coverage PacBio genomes obtained from a family trio, DRILR found an average of 247 non-reference L1 insertions per individual. By generating a consensus sequence at each insertion site this analysis had the capacity to resolve L1 insertions with a sensitivity for reference L1 s of > 95% and, detect TPRT hallmarks. This estimate of L1 genetic diversity was significantly higher than that obtained by other tools, and in prior analyses of short-read sequencing data, but was supported by a validation rate of 97%. These findings highlight the current and future promise of long-read sequencing technologies in finding novel L1 insertions.

Alice Lee (Boston Children’s Hospital, USA) presented an integrative analysis of gastrointestinal cancer genomes and matched RNA-seq profiles, identifying p53 mutation status and cancer immune activity as correlates of tumor-specific L1 retrotransposition rate. Known cancer genes, including ROBO1, were found with recurrent somatic L1 insertions. A tumor-specific L1 insertion was found to cause exon-skipping in the L1 repressor MOV10 and, strikingly, coincided with an unusually high load of L1 activity in the affected tumor. Similarly, in a Batten disease patient, a pathogenic SVA insertion was found to cause mis-splicing of the *MFSD8* gene. These findings highlight splicing abnormalities as an important mechanism by which retrotransposition in the germline and cancer can impact pathogenesis.

Geoffrey Faulkner (Mater Research Institute, University of Queensland, Australia) presented a novel method for highly multiplex bisulfite sequencing analysis of individual donor L1 loci. Combined with whole genome sequencing and targeted L1 sequencing of nearly 1000 human and mouse samples (primarily tumors, stem cells and individual neurons), this approach enabled them to study the methylation state of specific donor L1 s associated with de novo L1 insertions and identified by 3′ transductions. In particular, they focused on a somatic L1 insertion found in several hippocampal neurons isolated from one individual and stringently PCR validated. Intriguingly, the donor L1 for the insertion was slightly 5′ truncated, enabling its escape from repression. These results highlight how L1 retrotransposition is dynamically and specifically regulated in the soma.

## Evolution and regulation of mobile elements (Ruth Lehmann, session chair)

Cedric Feschotte (Cornell University, USA) highlighted two examples of convergent cooption of TEs. In the first example Feschotte described the role of a primate-specific endogenous retrovirus MRE41B in providing cis-regulatory sequence for a network of innate immune genes. In another and striking example he described Arc, a trans-cellular RNA signaling system derived from retrotransposon Gag proteins that modulates synaptic plasticity in flies and vertebrates.

Henry Levin (NICHD, National Institutes of Health, USA) described a direct test for how TE integration may wire regulatory networks by using regulatory sequences derived from transposable elements. Levin and colleagues created a library of LTR retrotransposon Tf1 insertions in the yeast *Schizosaccharomyces pombe.* Under stress condition and prolonged competition, Tf1 integration was beneficial to its host by creating a pool of new alleles, linked to TOR and other stress response pathways. Similarly, in wild isolates of *S. pombe* integration clustered next to genes important for sporulation efficiency and heat shock resistance, suggesting transposition plays an important role in adaptation in natural populations.

Tugce Aktas (Akhtar lab, Max Planck Institute of Immunobiology and Epigenetics, Germany) spoke about the potential evolutionary advantage conferred by Alu elements, which belong to the short interspersed nuclear element (SINE) family of repetitive elements that preferentially integrate into intronic regions of human genes. She found that the nuclear RNA helicase DHX9 neutralizes RNA processing defects caused by Alu invasion by resolving long dsRNA, thereby allowing proper RNA processing and nuclear export. DHX9 specifically interacts with the interferon-inducible isoform of ADAR. Aktas speculated that connecting ADAR, which carries dsRNA recognition domains, with the DHX9 helicase played a critical role in the innate immunity response to defend against viruses, as well as discrimination of self vs. non-self nucleic acids in multicellular organisms, and resolved problems caused by the host-generated dsRNA due to TE accumulation in genomes.

Della Fixsen (Elde lab, University of Utah, USA) discussed the role of horizontal gene transfer from host genomes as an ongoing source of genetic variation among viruses in the arms race between viruses and their hosts. Retrotransposition has been proposed as one possible mechanism of transferring host genes to viruses. In support, Fixsen presented results of a screen that argued that LINE elements are transferred from the host to poxvirus genomes.

Jason Fernandes (Haussler lab, University of California Santa Cruz, USA) demonstrated the UCSC Repeat Browser, a tool to map genomic data to consensus repeat elements, which can be used to trace “arms race” scenarios between KRAB Zinc Finger (KZNF) proteins and TEs. Using this tool in combination with large-scale ChIP-Seq studies of KZNFs, and with evolutionary analysis of TEs and KZNF binding sites revealed that as a TE mutates to escape from the grip of a KZNF, other KZNFs target separate motifs within the escaping element, forming groups of KZNFs that coordinate to repress specific TE families.

Manvendra Singh (Izsvak lab, Max Delbrück Center, Germany) reported on HERV(H), a human endogenous retrovirus. He proposed that in the pluripotent cells of the epiblast, HERV(H) copies act as functional enhancers to modulate neighboring gene expression and suppress mutagenic transposable elements. These studies also have clear implications for induced human embryonic stem cells, where new HERV(H) elements are not activated.

Ruth Lehmann (NYU School of Medicine, USA) reported on TE regulation during germline development in the fruit fly, *Drosophila melanogaster*. The piRNAs pathway regulates TEs specifically in the gonad by regulating RNA transcript levels. Lehmann and colleagues now identify splicing regulation as a new role for the Piwi pathway in protecting the genome against TE mobility. They show that splicing of specific introns of the P-element, a DNA TE, and the Gypsy retrotransposon are regulated via establishment of repressive chromatin states that rely on the function of the Piwi-piRNA complex proteins.

## Genome structural variation and neuroplasticity in Primates (Rusty Gage, session chair)

Evan Eichler (University of Washington, USA) discussed how human, chimpanzee, and gorilla show a preponderance of interspersed duplications (> 50%) when compared to other mammalian genomes where the duplications are primarily clustered. This architectural difference predisposes great ape genomes to large-scale copy-number microdeletions and microduplications. Ape duplications have accumulated non-randomly in time and space. Intrachromosomal ape expansions are associated with specific core sequences that appear to have duplicatively transposed segments of the genome in a lineage-specific manner. This property has led to large, gene-rich regions being radically restructured between closely related species such as chimpanzee and human. This potential to duplicate, shuffle and juxtapose diverse genic segments has led to the formation of novel genes that have evolved specifically within the human lineage. Functional data suggest they have contributed to unique neuroadaptive aspects of humans.

Chris Walsh (Harvard University, USA) presented data on whole genome sequence analysis of single neurons from human postmortem brain. They found that somatic LINE insertions are found in about one per two neurons, while each neuron shows 300–900 somatic point mutations per genome at birth, increasing to 2500 by age 80, and even higher numbers in dentate gyrus granule neurons. Analysis of the types of mutations can define those mutations that are likely to be congenital, i.e., formed during cell division, and which are acquired through the post-mitotic life of a neuron. Transcription and oxidation-induced damage appear to be important causes of mutations that occur in post-mitotic neurons.

Molly Hammell (Cold Spring Harbor Laboratory, USA) presented work exploring the role of the RNA binding factor TDP-43 in controlling TE expression. This contribution to the silencing of repetitive elements presents a novel function with potential implications for TDP-43 linked diseases. In particular, 90% of patients with the neurodegenerative disease ALS show some degree of TDP-43 aggregate pathology, suggesting that a substantial subset of ALS patients could show de-silencing of TE expression in the tissues that lose functional TDP-43 protein. To support this, Hammell showed evidence from expression data from two patient cohorts where a large fraction of ALS patients shows elevated levels of TE expression in motor cortex. Elevated TEs in these samples included young TEs with the potential for retrotransposition. These data suggest that aberrant expression of TEs could be an important component of TDP-43 mediated pathology in ALS.

Angela Macia (Muotri lab, UC San Diego, USA) explained that Aicardi-Goutières syndrome (AGS) is a developmental disease characterized by neuroinflammation with onset in early infancy. AGS arises when Three-prime repair exonuclease-1 (TREX1) is mutated. Macia and colleagues developed a human stem cell 3D cortical organoid model using iPSCs from patients with AGS. They found neurons suffered apoptosis and astrocytes produced an increased secretion of type-I interferon (IFN). Analysis of the cells showed an accumulation of LINE-1 retroelements, and the use of reverse-transcriptase inhibitors rescued the neurotoxicity observed in AGS cells in vitro, suggesting a potential use in therapy for AGS.

Using single-cell sequencing and machine learning, Rusty Gage (The Salk Institute, USA) showed that somatic L1-associated variants (SLAVs) are composed of two classes: L1 retrotransposition insertions and retrotransposition-independent L1-associated variants. He showed that a subset of SLAVs comprises somatic deletions generated by L1 endonuclease cutting activity. His lab demonstrated that SLAVs are present in crucial neural genes, such as *DLG2* (also called *PSD93*), and affect 44–63% of the cells in the healthy brain. He also reported that retrotransposition may represent a form of plasticity in response to maternal care in selected brain regions. Maternal care also alters DNA methylation at YY1 binding sites implicated in L1 activation and affects expression of the de novo methyltransferase *DNMT3a*. These observations indicate that early life experience can drive somatic variation in the genome via L1 retrotransposons.

## Transposition and gene regulation (M. Joan Curcio, session chair)

Joanna Wysocka (Stanford University School of Medicine, USA) discussed her work on the impact of TEs on host gene regulation. She first talked about the role of intronic L1 s located in transcriptionally permissive environments in negatively modulating host gene expression in human cells. This effect appears to be a collateral result of the epigenetic silencing mechanism specifically targeting full-length, euchromatic L1 s and is mediated by the HUSH/MORC2 complex. In the second part of her talk, she showed that during human preimplantation development, LTRs of another TE class, endogenous retrovirus HERV-K, function as long-range enhancers for host genes. To systematically perturb function of nearly 700 HERV-K LTR5HS insertions throughout the genome, Wysocka coupled a new method of guide RNA (gRNA) assembly termed chimeric array of gRNA oligonucleotides (CARGO) with CRISPR interference or activation (CRISPRi/a). Expression analysis confirmed that CARGO is indeed able to target LTR5HS elements en masse and identified 275 human genes reciprocally affected by LTRHS CRISPRa vs CRISPRi. Interestingly, effects of LTR perturbation on host gene expression occur over large genomic distances, consistent with the role of these elements as long-range enhancers in early embryonic cells.

Elizabeth H. Kellogg (Nogales lab, University of California, Berkeley, USA) described a collaboration with Don Rio’s lab to determine the structure of the P-element transposase using cryo-EM. They were able to show that the purified protein is active and in sufficient quantities for structural studies. Their structure so far indicates that the P-element transposase likely adopts a unique architecture compared to structures of other transposase superfamilies. With cryo-EM they have sufficient resolution to see that the P-element is a dimer, and can begin to visualize the bound P-element DNA.

Pascale Lesage (CNRS, Inserm, Institut Universitaire d’Hématologie, France) explained that In *S. cerevisiae*, Ty LTR-retrotransposons target their integration into regions with low coding potential to limit deleterious effects. Ty1 integrates preferentially in a 1-kb window upstream of Pol III-transcribed genes and targets nucleosomal DNA near the H2A/H2B interface. Lesage and collaborators previously showed that Ty1 integration site preference requires an interaction between integrase and the AC40 subunit of RNA Polymerase III. Lesage presented new data on the identification of a sequence in integrase that is required for the interaction with RNA polymerase III. Mutations of this sequence reduce Ty1 integration at tRNA genes and induce a redistribution of insertion events in subtelomeres, as observed previously with an AC40 loss of interaction mutant.

Robert Martienssen (Cold Spring Harbor Laboratory, USA) described a microRNA in plants, miR845, that recognizes the tRNA^Met^ primer-binding site of LTR-retrotransposons in *Arabidopsis* pollen. As a result, a 21–22 nucleotide siRNA accumulates and forms a hybridization barrier between diploid seed parents and tetraploid pollen parents (the triploid block). This is another case of small RNAs targeting primer binding sites to control retrotransposons.

Joan Curcio (Wadsworth Center, USA) described a recently published study performed in collaboration with Randall Morse (Wadsworth Center) in which they demonstrated that the Mediator co-transcriptional activator complex regulates Ty1 retrotransposition over a > 10,000-fold range by modulating the levels of an internal transcript, Ty1i RNA. Curcio suggested that this remarkable instance of host modulation of retrotransposition indicates that Ty1 activity might benefit the host under defined circumstances. As an example, she discussed work demonstrating that formation of the Ty1 virus-like particle assembly site influences spindle pole body inheritance, a determinant of genome plasticity.

## Workshop 2: The neurobiology of mobile elements (John Goodier, session chair)

Jasmine Jacob-Hirsch (Sheba Medical Center, Israel) provided evidence that relative to normal brain, somatic L1Hs insertions are more frequent in neurodevelopmental disorders, including Rett syndrome, tuberous sclerosis, ataxia-telangiectasia and autism. Most somatic brain L1 insertions were found in pre-existing repeats and appear to be endonuclease independent. Jacob-Hirsch proposed that such evolutionarily selected sites act as “lightning rods” that attenuate L1 mutagenic effects, a mechanism that may be breached in neurodevelopmental diseases.

Michelle Percharde (Ramalho-Santos lab, UC San Francisco, USA) reported that LINE-1 RNA is highly abundant in nuclei of mouse embryonic stem cells and embryos, and its expression is uncoupled from high levels of retrotransposition. LINE-1 RNA acts as a nuclear scaffold that recruits the chromatin factors nucleolin and Kap1 to regulate target genes. As a result, L1-depleted ES cells and embryos suffer significant self-renewal defects, highlighting the importance of LINE-1 expression in early development.

Caterina Gasperini (De Pietri Tonelli lab, Italian Institute of Technology, Genoa, Italy) reported on the P-element-induced wimpy testis (PIWI) RNA pathway in adult neurogenesis. The presence of piRNAs in the adult nervous system of mammals suggests the possibility they may regulate TE-dependent somatic mosaicism in mature neurons. However, low abundance of PIWI-pathway proteins and piRNAs raised concern for the relevance of this pathway in the CNS. Gasperini described that PIWI and piRNAs are particularly enriched in specific cell subpopulations of the adult hippocampus, in vivo. Manipulation of one PIWI pathway-protein altered differentiation of neural stem cells, in vitro.

Johan Jakobsson (Lund University, Sweden) also presented data demonstrating that ancient LINE-2 (L2) transposable elements generate microRNAs (miRNAs) that are highly expressed in human neuronal precursor cells and embryonic brain tissue. In turn, these transposon-derived microRNAs target and regulate genes carrying L2 sequence in their 3’UTRs. These data reveal a post-transcriptional miRNA regulatory network based on TEs active in human cells.

Julia Fuchs (Prochiantz lab, Collège de France, France) showed that oxidative stress triggers L1 overexpression and DNA damage in adult mouse dopaminergic neurons, a neuronal population that frequently degenerates in Parkinson’s disease. Anti-L1 strategies protected against acute oxidative stress-induced DNA damage and neuronal cell death in vitro and in vivo. It was hypothesized that age-related increase in L1 expression and reduced DNA repair induces DNA damage, which could contribute to age-related neurodegeneration.

Frank Jacobs (University of Amsterdam, Netherlands) described his research into how an evolutionary arms race between TEs and KRAB-Zinc Finger Proteins (KRAB-ZNFs) has re-shaped gene regulatory networks involved in human brain development. He presented findings on a KRAB ZNF that initially evolved to repress TEs, but has now become a repressor of genes central to brain development. These findings support a more general concept that the creation of new KRAB ZNFs added a primate-specific layer of gene regulation.

John Goodier (Johns Hopkins University School of Medicine, USA) discussed features of L1 ORF1 protein analogous to some neurodegeneration RNA-binding proteins, including formation of cytoplasmic and nuclear aggregates. Some ALS proteins bind and colocalize with ORF1p, and when overexpressed limit cell culture retrotransposition; these include TDP-43. Analysis of sporadic ALS-related RNA-Seq datasets and tissues failed to yield compelling evidence for global TE misregulation, although a previous RNA-Seq study showing TE expression altered in C9ORF72-associated ALS was further supported.

Zsuzsanna Izsvák (Max Delbrück Center, Germany) reported on a domesticated function of PiggyBac-derived gene 1 (*PGBD1*), which originated in Old World Monkeys as the fusion of transposase of a PiggyBac DNA transposon and a SCAN domain, a protein-binding motif frequently found at the N-termini of C2H2 zinc-finger proteins. While PGBD1 has no detectable transposase activity, it binds to exonic splice enhancer motifs near exon-intron boundaries as well as to transcriptional enhancers. PGBD1 possesses a differential binding pattern in neuronal progenitor cells (NPCs) and differentiated neurons. Curiously, SRPK2, a schizophrenia susceptibility factor, is a PGBD1 target in both NPCs and neurons. Collectively, the data suggest that PGBD1 has neuroprotective function.

## LINE-1 dances and transposon architecture (Jef Boeke, session chair)

John Moran (University of Michigan, USA) demonstrated that human LINE-1 RNAs occasionally undergo splicing and that retrotransposition of the spliced transcripts leads to the formation of Spliced Integrated Retrotransposed Elements (SpIREs). SpIREs account for approximately 2 % of annotated full-length primate-specific LINE-1 s and often lack critical sequences within their promoters. Thus, SpIREs represent evolutionary “dead ends”. Moreover, Moran demonstrated that DNA sequence changes within the 5’ UTR that enabled LINE-1 to evade the repressive effects of host factors lead to the generation of new LINE-1 splicing events and that retrotransposition of the resultant spliced mRNAs can generate new classes of SpIREs. Thus, these data demonstrate how genetic conflicts between L1 and the host genome have influenced LINE-1 expression and retrotransposition over evolutionary time.

Prescott Deininger (Tulane University, USA) previously showed that the nucleotide excision repair (NER) pathway plays a role in regulating L1 insertions. In a collaboration with Victoria Belancio, it was found that mice defective for NER showed this same regulation in vivo. They find that in patient cells that are defective for Transcription-Coupled NER (TCR), the typical bias in the orientation of L1 insertions in genes is eliminated. This suggests that TCR selects against sense-oriented L1 inserts in transcribing genes. Deininger and Belancio also discussed a pipeline for RNA-Seq analysis they used for specific L1 loci that detected differential expression in different normal tissues.

Gael Cristofari (IRCAN-INSERM/CNRS, France) expanded the use of ATLAS-seq to interrogate the distribution of de novo L1 retrotransposon insertions in the genome of cultured cells. L1 behaves differently from most transposable elements and retroviruses analyzed so far, in the sense that its insertions are only very modestly influenced by chromatin states. Remarkably, the distribution of these novel insertions differs significantly from that of endogenous insertions subjected to evolutionary selection. Cristofari also provided evidence that L1 retrotransposition in dividing cells is linked to host DNA replication.

Tao P. Wu (Xiao lab, Yale University, USA) discussed his characterization of the histone variant H2A.X. To identify properties of DNA at H2A.X-containing nucleosomes, Wu developed a novel approach of ChIP-Seq, “SMRT-ChIP” which can simultaneously determine DNA modifications and genomic sequences of H2A.X deposition sites. This approach combines Native Chromatin Immunoprecipitation (N-ChIP) and single molecule real-time sequencing (SMRT) technology. Wu described the unexpected discovery of N6-methyladenine (N6-mdA), a DNA modification not previously detected in mammals. He reported that N6-mdA specifically targeted young full-length LINE-1 retrotransposons in mouse ESCs. Moreover, they identified ALKBH1 as the demethylase of N6-mdA in mouse ESCs. Until this work, 5-methylcytosine and its derivatives were the only known functional DNA modifications in mammals. N6-mdA has the potential to impact the paradigm of mammalian epigenetics by adding another modification that could control LINE-1.

Jef Boeke (New York University, USA) discussed multiple experiments suggesting that the LINE-1 element life cycle targets the S phase of the cell cycle. He discussed evidence that the TPRT step of retrotransposition occurs during S phase. ORF1 protein and ORF2 protein show distinct localization dynamics, with ORF1 initially perinuclear but cytoplasmic, but subsequently and transiently (immediately after mitosis) present in newborn daughter cell nuclei as well. Nuclear localization disappears rapidly in early S phase as a consequence of nuclear export. Proteomics studies support this view and suggest that replication forks might represent a preferred target. He described evidence for at least two distinct populations of LINE-1 ribonucleoprotein particles (RNPs), a cytoplasmic RNP containing both ORF1p and ORF2p, and a nuclear RNP lacking ORF1p. A possible interpretation is that ORF1p delivers the ORF2/RNA complex to chromatin targets, exploiting a “window of opportunity” during mitotic nuclear envelope breakdown, leading to TPRT during S phase. This echoed Cristofari’s observations.

## Future directions

Groundbreaking technologies such as CRISPR will increasingly reveal the roles TEs play in early stages of development and in somatic transposition relating to neurobiology and cancer. New sequencing strategies capable of extremely long reads will inevitably determine the structure of highly repeated genomes. These frontiers in understanding the genome will undoubtedly lead to new questions that will be addressed in future meetings.

## St Malo 2020

The tradition of a large international meeting on ‘mobile DNA’ will continue. The International Congress on Transposable Elements (ICTE) will be held April 25–28, 2020 in St Malo, France. Stay tuned for more details!

## Abbreviations

AAV: Adeno-associated virus
ADARs: Adenosine deaminases acting on RNA
ALS: Amyotrophic lateral sclerosis
CARGO: Chimeric array of gRNA oligonucleotides
circRNA: Circular RNAs
CNC: Copy number control
CRISPR: Clustered regularly interspaced short palindromic repeat
DRILR: Detecting Repeat Insertions in Long Reads
dsRNA: Double-stranded RNA
EAGs: EER-associated genes
EERs: Editing Enriched Regions
gRNA: Guide RNA
HDR: Homology-directed repair
HERV-K: Human endogenous retrovirus K
ICTE: International Congress on Transposable Elements
IFN: Type-I interferon
KRAB-ZNFs: KRAB-Zinc Finger Proteins
KZNF: KRAB Zinc Finger
lnc: Long, non-coding
LTR: Long terminat repeat
miRNAs: microRNAs
N6-mdA: N6-methyladenine
Nbr: Nibbler
N-ChIP: Native Chromatin Immunoprecipitation
NER: Nucleotide excision repair
NPCs: Neuronal progenitor cells
*PGBD1*: PiggyBac-derived gene 1
piRNAs: PIWI-interacting RNAs
PIWI: P-element-induced wimpy testis
RNPs: Ribonucleoprotein particles
RTEs: Retrotransposable elements
SATAY: Saturated Transposon Analysis in Yeast
SHERLOCK: Specific high-sensitivity enzymatic reporter unlocking
SINE: Short interspersed nuclear element
SLAVs: Somatic L1-associated variants
SMRT: Single molecule real-time sequencing
SpIREs: Spliced Integrated Retrotransposed Elements
TCR: Transcription-Coupled NER
TEs: Transposable elements
TPRT: Target primed reverse transcription
TREX1: Three-prime repair exonuclease-1
Zuc: Zucchini

